# COVID-19: How did community pharmacies get through the first wave?

**DOI:** 10.1177/1715163520945741

**Published:** 2020-09

**Authors:** Paul A. M. Gregory, Zubin Austin

**Affiliations:** Leslie Dan Faculty of Pharmacy and the Institute for Health Policy, Management and Evaluation, Faculty of Medicine, University of Toronto, Ontario

## Abstract

**Background:**

The coronavirus disease 2019 (COVID-19) pandemic of early 2020 was one of the most impactful events in living memory. As an essential service, community pharmacies remained open to provide care and service. The unprecedented nature and scale of the pandemic triggered considerable change in daily practice. In anticipation of future pandemic waves and similar mass-scale civil disruptions, it is important to understand how community pharmacies adapted and responded in the early weeks of COVID-19.

**Methods:**

A combination of convenience, snowball and purposive sampling methods was used to recruit staff from community pharmacies across Ontario, from a variety of different practice locations and types. A semistructured focus group interview protocol was used to elicit experiences. Data gathering was undertaken until the point of saturation. Thematic analysis was used to surface common experiences and to describe how community pharmacies adapted and responded.

**Results:**

A total of 39 participants (pharmacists, registered technicians and assistants) from 11 different pharmacies participated in this study. Data were coded based on 1) what happened, 2) how community pharmacies responded, and 3) what worked and did not work to support pharmacy staff in continued provision of service and care. Key findings included the collapse of provision of nondispensing remunerated services, the central role of managerial decisions in supporting resilience (e.g., change to 8-hour shifts from 12-hour shifts) and the central role of technology in supporting continuity of quality pharmacy services.

**Discussion:**

With anticipated future pandemic waves, preparedness of community pharmacy will be essential. This study provides important insights based on participants’ own experiences regarding ways employers can better support staff in provision of care and service to patients during times of mass-scale civil disruption. *Can Pharm J (Ott)* 2020;153:xx-xx.

## Background

In mid-March 2020, the lives of virtually every Canadian and the practices within every community pharmacy across the country literally changed overnight. Several months earlier, a novel coronavirus infection in Wuhan, China, set off a chain reaction of events internationally that became the most impactful global disruption in living memory.^1^ On March 11, 2020, the World Health Organization (WHO) declared a global pandemic, triggering global population-wide lockdowns affecting billions of people.^2^ The magnitude of the COVID-19 crisis truly defied imagination—or preplanning—yet despite the scale and impact of the situation, community pharmacies across Canada remained open and continued to provide service and care to their patients. At the time this article was written, the full story of COVID-19 was still not known, nor is there any clarity as to whether or when any return to a discernible prepandemic “normal” will be possible.

In 2007, we published research examining previously unimaginable public health crises.^3^ This article focused on the ways in which community pharmacists in Ontario coped with severe acute respiratory syndrome (SARS) in 2003 and the massive disruption of the electrical transmission grid across eastern North America in 2005. The focus of this research was on the adaptation strategies community pharmacies used to maintain service despite the collapse of basic infrastructure (electricity, primary health care) and concluded that the profession needed to plan for and expect similar situations in the future because “emergencies and civil crises will continue to occur.”^3^

**Knowledge Into Practice**

- Mass disruption events, such as the coronavirus disease 2019 (COVID-19) pandemic, have tidal impacts on pharmacy practice; assessing how the profession adapts and responds is essential in order to better prepare for the next such event.
- In the early days of the COVID-19 pandemic, demand surges resulted in a collapse of delivery of remunerated clinical services as all attention and focus shifted towards drug distribution and supply chain–related activities. The impact on nonremunerated clinical services requires further evaluation.
- Managerial and organization-level supports for workforce resilience include scheduling practices to support continuity and recovery, as well as workflow designed to reduce multitasking and cognitive overload demands.
- Pharmacies that embraced and integrated communications and medication management technologies prior to the pandemic appeared better able to manage demand surges.
- Attentiveness to the pharmacy workforce’s safety needs (e.g., personal protective equipment, social distancing practices, additional security) is essential to support delivery of quality services and care.

Pandemic planning has been in place across provincial health systems for many years; a globally influential study by Cox, Tamblyn and Tam^4^ (who eventually went on to become Canada’s chief public health officer during the COVID-19 pandemic) described principles for system responsiveness, drawing upon lessons learned during SARS, particularly in Toronto. In particular, this study focused on the need to coordinate health care screening and delivery activities across diverse agencies, but with scant attention paid to the potential role and importance of community pharmacy. In 2008, Traynor^5^ noted that “pharmacists matter in pandemic response” and crafted a role for community pharmacies as decentralized primary care service hubs providing triaging, vaccination, public health screening and other services to relieve pressure on emergency rooms and family physicians’ offices. Fitzgerald et al.^6^ examined promising practices in pandemic planning with respect to optimization of the role of community pharmacies: at that time, they noted that, despite ongoing discussions and formal commitments, there were few effective examples of how to integrate privately owned pharmacies into a broader public health agenda. Nationally and globally, pharmacy organizations have developed statements and toolkits to provide guidance to community pharmacists during pandemics. FIP (the International Pharmacy Federation) recently published guidelines for pharmacists and the pharmacy workforce to manage COVID-19, highlighting core responsibilities associated with stewardship of the drug supply chain, patient education and provision of public health services where scope of practice permits.^7^ Initially produced in 2009, the Canadian Pharmacists Association’s “Pharmacists’ Guide to Pandemic Preparedness” focused on pragmatic issues, including strategies for safeguarding personal health and safety, initiation of infection control programs within retail environments and guidance for managing surges in patient demand and store-based traffic.^8^ Similar guidance documents have been produced by the Royal Pharmaceutical Society (United Kingdom)^9^ and the American Pharmacists Association,^10^ reflecting their unique national and local health system needs and resources.

While important and helpful, such documents and previous research could simply not predict the magnitude of what occurred in the late winter/early spring of 2020. Currently, there is general consensus that the evolution of COVID-19 will involve successive “waves” or cycles of peak infection followed by trough periods.^11^ In order to prepare better for what may be several years of disruption in society and professional practice, it is valuable to generate early data regarding how community pharmacists managed the first wave of COVID-19 in order to better support forward planning and preparation.

## Research objective

The objective of this research was to describe the experience of community pharmacies during the early first phase (mid-March to mid-May 2020) of the COVID-19 pandemic in Ontario, Canada. For the purpose of this research, the unit of study was the pharmacy as an organization, rather than individual pharmacists, technicians or other staff members.

## Methods

The highly unusual circumstances of the COVID-19 pandemic, coupled with the magnitude of its societal impact, introduced significant challenges and opportunities for addressing the research objective. Given the fast-changing nature of the situation and the absence of any real precedent for this kind of world-changing event, a qualitative, exploratory research method was selected in order to provide a starting point for future research. No guiding principles or theoretical framework currently in existence was sufficiently robust to apply to this pandemic situation, given its breadth and dimensions.^12^ Equally importantly, this unprecedented situation provided the researchers with an opportunity to engage in real-time research—actively engaging with participants as the pandemic situation itself was evolving rather than waiting for it to end and then retrospectively examining how pharmacists responded.^13^ The value of rapid-response real-time research in this context was significant, as it could potentially inform practice and policy decisions related to subsequent follow-up waves or surges of COVID-19 in the months or years ahead.^14^ As a result, a combination of convenience, snowballing and purposive sampling methods was used to recruit participants, using a method described by Yin.^15^

For convenience purposes, sites for this study were community pharmacies involved in the University of Toronto’s experiential education program. Purposive sampling was used to identify potential research sites across the province of Ontario (urban [city or town], rural, suburban) representing different geographical locations and business types (independent, chain, grocery, etc.). Designated managers or owners in these pharmacies were approached using email and informed of the study objectives and invited to participate. The unit of analysis for this study was the pharmacy itself, rather than individual staff members: as a result, participation was limited to pharmacies where 3 or more staff members representing different job types (pharmacist, manager, owner, regulated technician, unregulated technician, assistant, etc.) agreed to participate.

Snowball sampling was also used for participant recruitment; all participants in this study were invited to nominate or recommend another colleague or friend in another pharmacy who they thought might be interested in participating in this study—a research team member would then follow up to provide this individual/group with further information and an invitation to participate. Snowball sampling is useful for rapid expansion of the potential pool of research participants but can lead to overrepresentation of similar kinds of individuals, as research participants (in general) are more likely to recommend colleagues who are similar demographically or with respect to practice type to themselves.^15^

To optimize the value of this snowballing technique, purposive sampling was also applied to the participant pool. Purposive sampling provided the research team with a more direct opportunity to filter potential participants based on core demographic characteristics, such as location of practice (urban, suburban, rural, small town), nature of practice (chain, independent, grocery, big-box retailer) and prescription volume (low [<250/day], medium [250-500/day] or high [>500 day]), in an effort to more broadly align the participant pool with practice demographics of the profession in Ontario. Inclusion criteria for this study were 1) working a minimum of 24 hours/week within the pharmacy for a minimum of 3 years; 2) working in a patient-facing role within the dispensary (rather than front shop); 3) pharmacist, regulated pharmacy technician or dispensary assistant only (no students/learners); and 4) English speaking. The combination of these 3 forms of sampling allowed for rapid assembly of a sample that was approximate (although not necessarily statistically representative) of the profession in Ontario.

Since the unit of analysis for this study was the pharmacy—rather than the individual employee within the pharmacy—a focus group method was selected that involved simultaneous interviews of all participants within the pharmacy followed by open-forum discussion. Due to social distancing requirements in place at the time of this research, all focus group sessions were conducted using technology (Microsoft Teams) to keep the interviewer and each participant safe and socially distant from one another and to support recording of all sessions. The Teams platform also supported generation of transcripts of recorded meetings, which facilitated rapid analysis and coding of data. Importantly, the Teams platform stores video and transcript data in an encrypted format that is not possible for the research team to fully control. As a result, absolute security and confidentiality were not possible in this study. This study was deemed low risk through institutional ethics review, and this information was fully disclosed to all participants.

Focus group interview sessions were conducted pursuant to a semistructured interview protocol that evolved during the course of the study (see online Appendix 1 for the final version used). As the focus of this project was on rapid research, there was no pilot testing of the protocol. Similarly, given the extraordinary and unprecedented circumstances associated with the COVID-19 pandemic, there were no available previously validated instruments that were useful in this research context, nor was there any relevant theoretical framework to guide its development. The semistructured nature of the protocol facilitated significant opportunities to customize questions and approaches to the unique experiences of the pharmacy team being interviewed in order to capture the broadest and deepest data possible.

All transcripts were independently reviewed by 2 members of the research team, who then met to reconcile coding and analysis after each interview. Interviews were all conducted by the same person (PAMG) to enhance consistency of data collection. Interviews proceeded to the point of thematic saturation, the point at which no additional new information was being adduced from participants. No compensation was available for participants in this study. The study received expedited approval from institutional ethics review, in recognition of its potential value and importance to the pharmacy community in anticipation of the next wave of COVID.

## Findings and Discussion

A total of 11 teams consisting of 39 individuals (pharmacists, regulated technicians, assistants, designated managers, owners, full-time, part-time and locum staff) participated in this research (see Table 1). Findings for this study were broadly categorized into 3 areas: 1) What happened? 2) How did we respond? and 3) What worked and what didn’t work?

**Table 1:** Demographic characteristics of participants (*n* = 11 teams = 39 participants)

Code	Location	Nature of practice	Rx volume*	Participants (years in this practice)
Pharmacy 1	Suburban	Chain	High	Pharmacist (13 years) Assistant (9 years) Assistant (5 years)
Pharmacy 2	Urban	Chain	High	Pharmacist (7 years) Pharmacist (6 years) Assistant (9 years) Technician (3 years)
Pharmacy 3	Rural	Independent	Low	Pharmacist (16 years) Pharmacist (16 years) Assistant (14 years)
Pharmacy 4	Suburban	Grocery	Medium	Pharmacist (9 years) Pharmacist (4 years) Assistant (9 years) Assistant (6 years)
Pharmacy 5	Urban	Independent	Medium	Pharmacist (27 years) Pharmacist (11 years) Assistant (7 years)
Pharmacy 6	Suburban	Big box	High	Pharmacist (9 years) Pharmacist (7 years) Pharmacist (5 years) Technician (3 years) Assistant (7 years)
Pharmacy 7	Small town	Chain	Medium	Pharmacist (19 years) Assistant (9 years) Assistant (8 years)
Pharmacy 8	Small town	Independent	Low	Pharmacist (29 years) Pharmacist (3 years) Assistant (22 years)
Pharmacy 9	Suburban	Independent	Medium	Pharmacist (13 years) Pharmacist (13 years) Assistant (11 years)
Pharmacy 10	Urban	Grocery	Heavy	Pharmacist (6 years) Technician (8 years) Assistant (4 years)
Pharmacy 11	Suburban	Big box	Heavy	Pharmacist (5 years) Pharmacist (5 years) Technician (4 years) Assistant (4 years) Assistant (3 years)

* Low = <250 prescriptions filled per day; medium = 250 to 500 prescriptions filled per day; high = >500 prescriptions filled per day.

### 1. What happened?

The speed and magnitude of practice change that was experienced in community pharmacy was the dominant theme across all participants. The notion that “everything, literally everything seemed to change overnight” was echoed repeatedly and unanimously. Across all pharmacy teams, the following practice changes were highlighted: 1) surges in volumes of patients seeking renewals of prescriptions, health advice/counselling and nonprescription medications; 2) increases in drug shortages and increases in unpredictability of short supply items; 3) increased workload with respect to dispensing as policies designed to prevent stockpiling or worsening shortages reduced allowable dispensing quantities; 4) significant decrease/complete collapse in provision of documented and remunerated clinical services such as vaccinations and MedsChecks; 5) increase in unremunerated clinical interventions (such as prescription modifications and waived fees for prescription renewals); 6) significant decrease in patient-facing activities related to education/counselling; and 7) significant increase in nonclinical customer service activities such as explanation of drug shortage issues or changes in prescription renewal policies.

It was nonstop and just like a tidal wave. The work exploded, but it wasn’t good work, you know? It was explaining, apologizing, having to say you’re sorry about back orders or short renewals, that sort of thing. I wish it had been, I don’t know, you know like we were called on to do more clinical responsible things but nope, it was just about dispensing dispensing, dispensing and everything else went out the window it seemed. That’s too bad . . . I guess in a crisis you get to see what’s really important and for us, it looks like it’s the dispensing not the clinical that’s seen as important. (Pharmacy 6)

You know, I’m actually amazed we did as well as we did. It was all hands on board, especially those first few weeks while we were just getting used to it all. I have never been busier in my life but I’m proud of how we all pulled together and we made sure everyone got what they needed. And we got so much appreciation from our customers—that really helped. . . . Sure, a lot of the things we used to do—MedsChecks . . . you just couldn’t any more, even basic counselling. But at the end of the day people needed to get drugs in their hands—or delivered at home!—and that’s the priority and we got that done so everyone should be proud we stepped up and made it happen. (Pharmacy 9)

Across participants in this study, there was consensus on the experience of practice during pandemic: prioritization of dispensing and drug distribution activities over virtually all other, particularly remunerated clinical activities. There was no consensus, however, on the significance of this common experience: some participants lamented this “backsliding” away from clinical work, while others expressed pride that they were able to provide what they thought to be the most essential of pharmacy services with minimal disruption to patients. It is unclear whether this situation is unique to Ontario (which was particularly hard-hit by the COVID-19 pandemic). However, given the general belief during this time that lack of credible information was a significant problem for the general public, the inability of community pharmacy to step up and serve a more clinical role in the community raises further questions regarding lost opportunities for the profession.

### 2. How did we respond?

Across all participants, there was broad agreement that the first 2 to 3 weeks following the WHO declaration of a global pandemic were the most challenging in their professional experience, given the lack of preplanning at the pharmacy or organizational level. Participants vividly described personal and professional fears associated with lack of information regarding the pandemic and in particular issues related to personal safety, lack of personal protective equipment (PPE) and lack of clear policies or practices to guide daily work. Pharmacists in particular commented on the lack of a central, credible repository of pharmacy-specific guidance that they could use to manage evolving questions or concerns. For example, when information regarding the potential value of hydroxychloroquine in reducing risk associated with COVID-19 was being promoted by some individuals, or when questions regarding the role of ibuprofen in worsening COVID-19 symptoms arose, pharmacists needed rapid access to high-quality information and found this difficult to find, highlighting a potential opportunity for future development.

For me, it all seemed to come down to information and communication. Of course, everyone understood that this was new and fast changing but what we needed—but never got, well, not in the first month or two at least—was clear direction, explanation or even just a clear “we don’t know” statement as to how to deal with different practical situations. It’s one thing [for management] to say “we don’t know” and that’s fine, but don’t just leave it at that. . . . We need something, otherwise it’s every man for himself, there’s no consistency and it . . . well, you saw what happened, it was disorganization and chaos. I hope we can do better the next time now that we know what happens if we just leave it to each store or pharmacist to decide things for themselves. (Pharmacy 7)

Of interest, national associations (such as the Canadian Pharmacists Association) had made availability of such information a key priority during this time, and its website and social media were updated frequently with best possible scientific evidence. Lack of awareness of this important repository of information by pharmacists in this study suggests that further work in either communicating or disseminating the availability of such information, or further training of pharmacists to locate credible available evidence, may be required.

Another common theme across all participants was the need to enhance pharmacy-based security, navigation and PPE practices. In particular, given high levels of public anxiety and uncertainty regarding the integrity of the drug supply chain in Canada, many participants reported difficult, sometimes frightening, interactions with members of the public and their desire for dedicated security to provide support and conflict management. The additional stress of these issues further heightened workplace pressures and reduced the capacity of all pharmacy team members to manage increasing demands. Most participants also highlighted the impact of inconsistent guidance and practices regarding PPE in the workplace—for staff and for customers/patients. While scientific consensus on the importance of masks or gloves had been inconsistent and changing, virtually all participants in this research reported a high level of anxiety around interactions with unprotected patients and customers and other staff, as well as a strong desire for greater clarity and leadership from employers and organizations around PPE and security issues. While pharmacy-based navigation (e.g., “safety bubbles” on floors to indicate a safe 2-metre zone, or directional wayfinding arrows to prevent bunching of people in aisles) became commonplace retail strategies in the second and third months of the pandemic, they were not widely used in the first few weeks, and this increased anxiety and concerns for pharmacy staff.

It was kind of weirdly unforgivable. I mean here we all are, the pharmacists, the team, and we’re stressed and we’re scared, we don’t have the right [PPE], the customers are freaked out with drug shortages and things. And now I’m expected to also be a security guard. I mean, that’s just not fair. I know how to handle patients when they are upset or angry but this was way beyond that. I don’t understand—I mean, it would have been so simple and yes maybe cost a bit of money—but having a dedicated security guard or someone who could help when things got heated, so the pharmacy staff aren’t feeling threatened or afraid, especially because, well, who knows if the person shouting or crying has COVID? (Pharmacy 5)

A final common theme across all participants related to the central role of workplace/employer support in enhancing personal resilience strategies. Participants in this study ranged considerably in terms of their years in practice, yet all were “experienced” and had developed strategies for managing time and interpersonal pressures associated with pre-COVID-19 pharmacy practice. Most participants noted that, despite this experience and a personal repertoire of effective coping and adaptation resources, the dimensions of the pandemic clearly meant that resilience was more than a personal issue: workplace policies were substantially more meaningful and integral to the ability of the pharmacy to actually function and for individuals employed within that environment to personally cope.

Before the next wave or cycle or whatever . . . we have to get ready, be better prepared. There are things I know I can do differently as a pharmacist, sure. But at the end of the day, what I learned was the most important thing is the way the whole operation manages, the way the whole store, or organization gets itself ready. These things . . . well, even if there never is a second wave or cycle, these changes in the way pharmacies run should just become routine because in the end, that’s going to make the pharmacy run better, be more efficient and actually have a greater impact for patients. And make it a better, happier place to work for the staff. (Pharmacy 4)

### 3. What worked—and what didn’t work?

As noted above, a common theme across all participants related to the importance of “organizational resilience” as opposed to personal coping strategies as a way of managing the demand surges and other stresses associated with the pandemic. While each practice has unique needs and issues, a series of common managerial strategies were identified that highlight the ways in which a thoughtful, deliberate and planned organizational response could enhance both pharmacy operations and personal resilience of staff. Key strategies identified by participants in this study included the following:

#### A. Shift length

Participants in this study were unanimous in noting that the length of time spent in the pharmacy in a patient-facing role was of considerable importance in predicting operational success and supporting personal resilience. Longer shifts (12, 14, or even longer consecutive hours worked) produced cognitive and emotional overload, which led to knock-on effects and delayed recovery. While historically some pharmacists may have preferred longer but fewer worked shifts per week for personal or family reasons, from an operational and psychological perspective, there was consensus among participants in this study that this was problematic. The need for recovery time following a shift, coupled with the physical and emotional exhaustion associated with such long shifts, was considerably exacerbated due to pandemic-related demand surges.

#### B. Scheduling patterns

Participants in this study highlighted the value of scheduling practices that focused on teams of pharmacists, regulated technicians and assistants, rather than on each person as an individual. Historically, many pharmacies have defined the individual worker as the unit of scheduling, rather than a team or line-based approach. The advantages of scheduling the same people to work in as consistent a manner as possible included greater efficiency in communication and interaction among team members (as there was no learning curve for collaboration at the start of each shift), as well as greater social comfort. Importantly during the pandemic, consistent scheduling also reduced the number of different people individual staff members would interact with over the course of a week or month of work, and this provided significant comfort. Finally, working with the same team consistently also made it easier for individuals within that team to provide social and psychosocial support for one another and to notice if any individual within that line/team was experiencing problems.

#### C. Reduction of multitasking

In an effort to reduce crowding in tight dispensary spaces, some participants noted their workplaces modified existing practices during the pandemic to support greater focus and specialization of work. For example, small counselling rooms (which were not used due to social distancing concerns) became separated office space the pharmacist could use to focus on a series of tasks (e.g., follow-up calls with patients or prescription verification) without the usual interruptions that occur within a dispensary environment. The positive effects of focus and reduction of multitasking were described as immediate and significant: the reduction in cognitive load associated with task focus was seen as not only important from a personal resilience perspective but also from a quality of care and clinical outcome perspective. Pharmacists who experienced this during the pandemic strongly supported the notion that this was a broader practice change that should become permanent postpandemic. Regulated technicians noted that the physical separation of the pharmacist from the dispensary not only allowed them to practise to a fuller scope, but it also permitted greater division of responsibilities that enhanced overall operational efficiency, while allowing the pharmacy team as a whole to actually enhance productivity, quality and workplace satisfaction.

#### D. Enhanced use of technology

Pharmacies across Canada are at various stages of embracing and integrating technology into daily practice. Prior to the pandemic, some pharmacies had access to sophisticated automated refill systems, email or videoconferencing capabilities and other technologies common in many other industries. Participants in this study, regardless of their role, noted that pharmacies that had embraced and integrated technologies more fully into daily practice prior to the pandemic were much better placed to respond and be resilient as the pandemic evolved. They noted that, while the learning curve to use such technologies can be challenging and steep, once learned, such technologies provided pharmacy team members with much greater control over day-to-day tasks. Technology provided pharmacy team members with options for contacting patients and prescribers and for prioritizing and managing workflow more effectively. Pharmacies that had not adopted technologies prior to the pandemic reported struggling with conventional routes of communication and interaction as demand surges peaked and as anxiety rose for patients; trying to integrate new technologies (e.g., Zoom) in the midst of the pandemic was also described negatively due to cognitive overload. Participants in this study noted that a desired outcome following the first wave of the pandemic would be greater investment in and more wholesale integration of diverse communications, monitoring and documentation technologies into their practices, which could further reinforce division of labour between pharmacists and regulated technicians, support greater task focus and provide more control over day-to-day work.

#### E. The need for more nonprofessional support staff

A final common theme was the problem of being “penny-wise, pound-foolish” during the pandemic with respect to staffing levels and staffing mix. Pandemic-triggered demand surges produced extraordinary levels of stress and increased workload, some of which could have been alleviated through additional hiring of casual staff members to perform nonregulated work, ranging from deliveries to inventory receiving to pharmacy-based security and wayfinding. Participants reported that many pharmacies appeared slow or hesitant to bring in additional staff or increase existing staffing hours, and as a result, professional staff were required to absorb increased nonprofessional workload, which further compounded workplace stress (e.g., regulated technicians driving around town delivering medications when they could be more useful managing refills, or pharmacists instructing customers on which way to walk down aisles to support social distancing). Much of the pandemic-induced demand surge was caused by nonpharmacy-related issues, and without additional nonprofessional support staff (e.g., drivers, receivers, greeters to hand out PPE or do front-door COVID-19 screening), this was simply added work that increased stress for everyone.

You know, we’ve learned so much in our pharmacy about how we should have done things differently and I hope that, well, we will put this all into place before the next wave hits. Simple things like, you can’t ask people to work 12-hour days when they have kids doing home schooling, or let’s schedule a group to work together so they can work as a team instead of individuals. It’s a no-brainer to just hire a temporary driver or someone to do deliveries so the technician can stay in the store and actually do that job. (Pharmacy 1)

At the time this article was written, the first wave of the pandemic appears to be subsiding in many (but not all) parts of Canada. Understandably, pharmacists and technicians, like the rest of the population, yearn for a return to the normalcy and predictability that existed on March 12, 2020. Public health officials caution us that this is not only premature, but it may be unlikely and unrealistic. Instead, as the future history of this time evolves, we may look at the first half of 2020 as a dry run for a future model of pharmacy practice. Findings from this study suggest that personal resiliency in times of a pandemic is built upon a foundation of organizational support and managerial strategies that help the workforce manage surge demands, unpredictability and stress. A shift away from multitasking towards task focus and more rapid integration of communication and medication management technologies in practice can help reduce cognitive overload that compromises both quality care and workplace satisfaction. Pharmacy owners and managers have the opportunity to materially impact the efficiency and effectiveness of their practices by shifting away from a traditional multitasking dispensing focus towards one that more fully embraces division of labour and supports regulated technician scope of practice.

### Limitations

It is important to note that the findings of this study must be interpreted with caution. As rapid response research designed to capture a fast-changing event in real time, the use of purposive, convenience and snowball sampling methods means the participant pool for this study was neither representative of the profession, nor can findings be considered generalizable beyond the sample frame. Rapid response research design also means triangulation and confirmation of participants’ statements and claims were not undertaken beyond the thematic saturation process previously described. Further, the focus group method, while useful in conceptualizing pharmacy staffing as a team, rather than individual, process, introduces social bias issues, which means individual participants may not have been as honest or forthcoming as they might have been in individual interviews. Despite these limitations and as a first approximation of examining community pharmacies’ response to a pandemic crisis, this study provides a useful starting point for discussion and reflection on preparation for an uncertain future.

## Conclusion

It is reasonable to believe that no one could have predicted the speed, severity, depth and magnitude of the COVID-19 pandemic. In a recent editorial, Farrell and Tsuyuki^16^ highlight the extraordinary efforts community pharmacists across Canada have made in stepping up to support their communities. These stories need to be promoted to reinforce public perception of pharmacy as an essential service during times of mass disruption events. Community pharmacy—like the rest of society—struggled to cope but learned important lessons for whatever comes next with this pandemic . . . or the next unrelated societal crisis. Rapid response research such as this, while imperfect, helps the profession better understand itself and prepare for the future, whatever that might hold. By conceptualizing resilience as both a personal and organizational process and highlighting specific strategies that may in the future help community pharmacy adapt more rapidly to the unexpected, findings of this study also help us to identify important opportunities to simply enhance the quality of practice and workplace satisfaction for employees within the profession. ■

## Supplemental Material

945741_Appendix_1_online_supp – Supplemental material for COVID-19: How did community pharmacies get through the first wave? Click here for additional data file. Supplemental material, 945741_Appendix_1_online_supp for COVID-19: How did community pharmacies get through the first wave? by Paul A. M. Gregory and Zubin Austin in Canadian Pharmacists Journal / Revue des Pharmaciens du Canada
