# Supplementary material for: COVID-19: How did community pharmacies get through the first wave?
Source: Can Pharm J (Ott). 2020 Aug 14;153(5):243–51. doi: 10.1177/1715163520945741 (PMC7429913; doi:10.1177/1715163520945741)
Supplement: 945741_Appendix_1_online_supp – Supplemental material for COVID-19: How did community pharmacies get through the first wave? [file 945741_Appendix_1_online_supp.pdf]

APPENDIX 1 Semi-structured interview protocol with sample quotations and coding framework

**Question**	**Sample transcript excerpt**	**Analysis and Coding**
Can you tell me about yourself and your pharmacy? How long have you been working here, what was a typical day like before COVID, how many prescriptions would you fill?	Well, we are a pretty busy place. We have 5 pharmacists – not all full time – 2 regulated pharmacy technicians and a few assistants. We also have students. This is one of the busiest stores in the [organization]. We'd fill more than a thousand prescriptions on a typical day. I've only been here 5 years but I'm probably the most senior person now. (Pharmacy 11)	High volume
I'm going to divide the last few months into specific periods of time to ask you some questions. Can you tell me what happened in your practice around March 12 when the pandemic was declared?	I don't think anyone really thought too much about it. Well, if you had kids in school, you mainly just thought about what you were going to do with them since school was cancelled for three weeks. I don't think any of us really thought much about the impact on the business or the store. So no one really – well, I wouldn't even know where to look for information about what we should have done in a pandemic. (Pharmacy 8)	Lack of information
What happened in the first week after March 12?	It was a disaster. Patients immediately wanted to stockpile especially with [respiratory medications] since of course they knew this was a respiratory thing. So the phone is ringing, the crowds are building, everyone was anxious. I've never seen it busier in my life. (Pharmacy 7)	Stockpiling<br>Increased workload
How did your practice respond in that first week after March 12? What cognitive/clinical services were you providing?	We had to scramble, I mean it was crazy. The dispensing workload went through the roof, we were like a machine. There was no chance to do anything else. I mean who wants to sit with a patient in a small room and do medschecks, or administer a vaccine or anything like that? Who'd be crazy enough to do that? It was just dispensing dispensing dispensing. (Pharmacy 5)	Drop in clinical services provision<br>Increased dispensing workload<br>Fear

What worked well that first week? What didn't work so well?	We should have known – well someone should have known – the workload was berserk, right? We needed more staff – not pharmacists but just more hands on board, right, to manage the crowds and deal with all the craziness. (Pharmacy 1)	Insufficient staffing<br>Insufficient planning<br>Insufficient resources
Let's move ahead to the following 2 or 3 weeks when the scale of COVID was clearer to everyone. What happened next in your pharmacy?	Well, at this point I'd say it was full blown panic here. We didn't know what to do about [PPE] for ourselves or our customers, everyone was freaked out. I mean the abuse – literal abuse, shouting, threats – it was terrible. I'm not a policeman, right, but I'm dealing with really scary people. I get it, everyone is freaking out but on top of that, I was honestly really scared for my own safety. (Pharmacy 4)	Fear<br>Insufficient resources
What worked well during this time? What didn't work so well?	At this point we all said, you know, we can't keep doing 12 or 14 hour shifts that was just too stressful too tiring. We figured it out and we were able to move to 8 hours and I think literally that's what saved it for us. We could actually breath. (Pharmacy 10)	Scheduling change
Let's move ahead to the next few weeks, the middle of April. What happened next in your pharmacy?	I'm so grateful we have (a telephone and computer-based renewal service). I hated it at first but it's a godsend during times like this. It really helped smoothe out the workload and gave us more control over things. With refills skyrocketing and everyone freaked out, it just gave us a sense of more control over things. (Pharmacy 11)	Technology
What worked well during this time? What didn't work so well?	The manager changed things so the pharmacists could use [the counselling space] like an office so all the initial review and order input and all the calls and things – it was great. It gave them space to think and no interruptions and honestly, I think it was essential. I hope it remains after this is done. (Pharmacy 11)	Task-focus
What do you think have been the most important things you've learned about managing during a pandemic?	I always thought I was pretty resilient you know? But a situation like this – it's got nothing to do with me as person or you. It's got everything to do with management, with the organization and them setting you up to	Resilience is organizational not personal<br>Sufficient resources

	succeed rather than fail. I'm so grateful [my organization] they got that, they provided the PPE, the extra staff we needed, they had the technology, right? (Pharmacy 4)	
Is there anything else you'd like to share that you think might be helpful?	It scares me but I think a crisis like this shows you that the bigger [a pharmacy organization is] the better of you are. Big organizations have money for things and can absorb more. I think this might be the end for many of the independent guys. (Pharmacy 5)	Insufficient resources

Gregory PAM, Austin Z. COVID-19: How did community pharmacies get through the first wave? Can Pharm J (Ott) 2020;153. DOI: 10.1177/1715163520945741.
